# Mesenchymal Stem Cells Derived Extracellular Vesicles Alleviate Traumatic Hemorrhagic Shock Induced Hepatic Injury *via* IL-10/PTPN22-Mediated M2 Kupffer Cell Polarization

**DOI:** 10.3389/fimmu.2021.811164

**Published:** 2022-01-12

**Authors:** Yunwei Zhang, Xiaofei Zhang, Hongji Zhang, Peng Song, Wenming Pan, Peng Xu, Guoliang Wang, Ping Hu, Zixuan Wang, Kunpeng Huang, Xiaodong Zhang, Hui Wang, Jinxiang Zhang

**Affiliations:** ^1^ Department of Emergency, Union Hospital, Tongji Medical College, Huazhong University of Science and Technology, Wuhan, China; ^2^ Center for Translational Medicine, Union Hospital, Tongji Medical College, Huazhong University of Science and Technology, Wuhan, China; ^3^ Department of Surgery, The Ohio State University, Columbus, OH, United States; ^4^ Department of Breast and Thyroid Surgery, Ningxia hui Autonomous Region People’s Hospital, Yinchuan, China; ^5^ Department of Emergency Surgery, Union Hospital, Tongji Medical College, Huazhong University of Science and Technology, Wuhan, China; ^6^ Department of Hepatobiliary Surgery, Union Hospital, Tongji Medical College, Huazhong University of Science and Technology, Wuhan, China; ^7^ College of Life Sciences, Wuhan University, Wuhan, China; ^8^ Department of Medical Genetics, Basic School of Tongji Medical College, Huazhong University of Science and Technology, Wuhan, China

**Keywords:** traumatic hemorrhagic shock, extracellular vesicle, hepatic injury, IL-10, Kupffer cell polarization

## Abstract

Traumatic hemorrhagic shock (THS) is a major cause of mortality and morbidity worldwide in severely injured patients. Mesenchymal stem cells (MSCs) possess immunomodulatory properties and tissue repair potential mainly through a paracrine pathway mediated by MSC-derived extracellular vesicles (MSC-EVs). Interleukin 10 (IL-10) is a potent anti-inflammatory cytokine that plays a crucial role during the inflammatory response, with a broad range of effects on innate and adaptive immunity, preventing damage to the host and maintaining normal tissue homeostasis. However, the function and mechanism of IL-10 in MSC-mediated protective effect in THS remain obscure. Here, we show that MSCs significantly attenuate hepatic injury and inflammation from THS in mice. Notably, these beneficial effects of MSCs disappeared when IL-10 was knocked out in EVs or when recombinant IL-10 was administered to mice. Mechanistically, MSC-EVs function to carry and deliver IL-10 as cargo. WT MSC-EVs restored the function of IL-10 KO MSCs during THS injury. We further demonstrated that EVs containing IL-10 mainly accumulated in the liver during THS, where they were captured by Kupffer cells and induced the expression of PTPN22. These effects subsequently shifted Kupffer cells to an anti-inflammatory phenotype and mitigated liver inflammation and injury. Therefore, our study indicates that MSC-EVs containing IL-10 alleviate THS-induced hepatic injury and may serve as a cell-free therapeutic approach for THS.

## Introduction

Severe trauma, especially traumatic hemorrhagic shock (THS), is a leading cause of mortality and morbidity in patients under 45 years of age and causes 5 million deaths each year worldwide (1). THS causes primary damage to organs, including hypo-perfusion of the main organs such as the liver, heart, kidneys, central nervous system (CNS), and lungs, and robust immune response activation. Subsequent reperfusion with resuscitation usually further triggers systemic inflammatory response syndrome, which may further aggravate tissue/organ damage, leading to multiple organ failure and even death (2, 3). THS-induced injury is accompanied by immune dysfunction exhibited by excessively inflammasomes release and multiple inflammatory signaling pathways activation. These events lead to an immune-suppressive state and host defense failure, eventually giving rise to inflammation-mediated infection and organ dysfunction (2, 4). Therefore, clinical treatments to block immune disorders and prevent organ damage are urgently needed and will have a significant global impact on patient outcomes after THS.

MSCs are adult stem cells that exhibit self-renewal, multi-linage differentiation and immune-modulation abilities (5). Previous studies showed that MSCs protect against liver and kidney injury by increase the release of anti-inflammatory cytokine such as IL-10 after hemorrhagic shock (6). However, affected by the purity, consistency, efficiency, scalability and other aspects of MSCs, its clinical application remains limited (7). Since MSC cannot be widely used in clinical practice and MSC could exert anti-inflammatory effects through IL-10, can supplementation of exogenous IL-10 suppress the inflammatory response of THS?

IL-10 is a potent anti-inflammatory cytokine that plays a crucial role during the inflammatory response, preventing damage to the host and maintaining normal tissue homeostasis (8). Previous studies showed that MSCs overexpressing IL-10 increase mitochondrial autophagy and protect against neuronal damage induced by traumatic brain injury (9). Notably, the clinical therapeutic effect of exogenous IL-10 is also limited because of its short half-life and instability *in vivo (* 10). Therefore, we explored the use of a more stable, specific and direct delivery system for MSCs-derived IL-10 to injured tissues.

Extracellular vesicle (EV) are small membrane-bound vesicles with a diameter of 30–1000 nm formed in a particular population of endosomes, called multivesicular bodies (MVBs), by inward budding into the lumen of the compartment (11). EVs are released into the extracellular matrix through their fusion with the cell membrane and secreted from various cell types under normal and pathological conditions (12). Notably, EVs can carry complex cargo molecules, such as proteins and RNAs, and transmit this information to neighboring cells to modulate immune response, apoptosis, angiogenesis and inflammation (13–17). MSC-EVs have exerted promising cell-free effects on tissue remodeling and functional recovery by delivering cargo molecules to recipient cells with better directionality and effectiveness (18, 19). Multiple reports showed that MSC-EVs exert anti-inflammatory effects in various inflammation-related diseases by delivering miRNAs and immunomodulatory proteins to macrophages (20–24). However, the potential physiological significance of MSC-EVs derived IL-10 in THS-induced hepatic damage remains unclear.

In this study, we investigated the potential role of IL-10 in the MSC-mediated protective effect in THS-induced hepatic injury and explored the possibility of EVs as IL-10 carriers. We found that IL-10-deficient MSCs lost protective function against THS-induced hepatic injury compared with IL-10 WT MSCs. Further experiments indicated that MSC-EVs carrying IL-10 as cargo alleviated hepatic injury. MSC-EVs were mainly taken up by liver resident macrophages, facilitating PTPN22 levels and consequent anti-inflammatory macrophage polarization. These findings implicate a role for IL-10 transported by MSC-EVs in mediating immunosuppression in THS-induced hepatic injury.

## Materials and Methods

### Animals

Male C57BL/6 and IL-10^ob/ob^C57BL/6 mice (8–12 weeks old, 20–25 g body weight) were purchased from HFK Bioscience CO, LTD (Peking, China). Mice were housed in the Laboratory Animal Center, an environment with controlled light (12 h light/dark), humidity (60 ± 10%), and temperature (23 ± 2°C), with food and water available ad libitum. Mice were fasted for 12 h before the start of the experiment. All experimental procedures involving animals were approved by the Animal Care and Use Committee of Wuhan Union Hospital.

### Establishment of the Mouse THS Model

Fixed volume shock simulates the body hemodynamic and intrinsic compensatory response to better levels compared with other shock models (25). We established an improved THS model based on fixed volume shock that was described previously (26). In brief, mice were anesthetized using sodium pentobarbital (60 mg/kg i.p.) and fixed on the table of a laminar flow operating room. After complete exposure of the left groin area by skin incision, the femoral artery, vein and nerve were separated without injury. The distal femoral artery was ligated using sutures (~1 cm in length), and a slipknot was tied at the proximal end of the artery. Between the two knots, PE-10 catheters for puncture were fixed with two sutures. The femoral artery was dampened by 2% lidocaine, and the right femur was fractured using two forceps forced in opposite direction until a fracture sound was heard, followed by holding for another 15 s. The fracture was subsequently confirmed by the abnormal movement of mice. The exposed left femoral artery was incised to a spindle-shaped cut with microscissors. After untying the proximal ligation, the prefixed PE-10 catheter was placed to let blood flow for approximately 10–15 min at a volume of 0.025 mL (every 1 g body weight) (27). After the surgery, mice were monitored for 90 min in an incubator at 32°C and then resuscitated with normal saline to three times their shed blood volume. For intervention, each mouse was administrated with MSCs (5×10^5^), EVs (20 μg), or rmIL-10 (50 μg/kg, R&D, MN, USA) through the femoral artery during resuscitation. All post-injury animals received subcutaneous injection of 0.1 mg/kg buprenorphine for analgesia (every 12 hours last for at least 72 hours) post-surgery.

### Isolation, Identification and Culture of MSCs

A mouse was sacrificed and soaked in 75% alcohol for 5 min. The epiphyses were separated and cut at both ends of the femur and tibia, and the femur and tibia were rinsed repeatedly with IMDM medium containing 10% fetal bovine serum. The flushed liquid was collected into a 50 ml centrifuge tube until the bone was white. The collected cell suspension was passed through a 70-micron filter and then centrifuged at 100 g for 10 min. The supernatant was discarded, and the cells were resuspended in IMDM medium containing 10% fetal bovine serum and inoculated into a flask. The cells, considered primary MSCs, were cultured in 37°C cell incubator with 5% CO_2_. The medium was changed on the following day and the cells were passed when they achieved 70%–80% confluency. The cell culture contained a variety of non-MSCs until the fourth generation of cells. After the fourth generation, the cells were purified into MSCs. MSCs of 4–10 generations were used in this study. MSCs at passage 4 were subjected to multilineage differentiation. The same passage numbers of WT and IL-10 knockout MSCs were used for individual experiments. Chondrogenic differentiation, adipogenic differentiation and osteogenic differentiation were evaluated using the Mouse Mesenchymal Stem Cell Functional Identification Kit (R&D, SC010), according to the instructions. In some experiments, MSCs were pretreated with 5 μg/mL GW4869 (Sigma, St Louis, MO, USA) or the same dose of DMSO (Paso Robles, Santa Cruz, CA, USA) for 12 h.

### Assessment of Liver Function

After the mice were anesthetized, approximately 800 μl blood from the apex of heart were collected. The blood was centrifuged at 300 g for 15 min at 4°C. Supernatants were collected for analyses using the LDH (A020-2), ALT (C009-2), and AST (C010-2) kits, all purchased from Jiancheng Bioengineering Institute (Nanjing, China). Plasma LDH, ALT and AST levels were measured using a spectrophotometer (ELx800, BioTek, USA).

### Isolation of Liver Non-Parenchymal Cells (NPCs)

Liver NPC isolation was performed as described previously (28). Briefly, mouse livers were perfused through the portal vein with 0.05% (w/v) collagenase IV (1 mg/mL; Sigma Aldrich, St. Louis, MO, USA) at 99 mL/h at 37°C. The livers were slightly massaged to help collagenase disperse into the entire organ. Livers were bluntly separated and centrifuged at 300 g for 11 min at room temperature. The supernatants were collected and mixed with OptiPrep separating solution (OptiPrep: phosphate-buffered saline=1:2, Sigma-Aldrich) and then density gradient centrifuged at 22°C, 400 g for 16 min to obtain liver NPCs.

### Bone Marrow Cell Separation

Bone marrow cells were isolated from mouse upper limbs. After removal of muscles and washes with 70% ethanol, limbs were removed, and the bone marrow cavity was rinsed with PBS. The mixture was centrifuged at 300 g for 10 min. The obtained precipitates were resuspended and incubated with red blood cell lysis buffer for 10 min. Bone marrow cells were obtained after centrifuging at 300 g for 10 min. Cells were counted and resuspended at a concentration of 5×10^6^/mL.

### Isolation of MSC-EVs

MSC-EVs were isolated as previously described (29). In brief, MSCs were cultured to a density of 2×10^7^ cells/T75 flask. Culture media were removed and cells were washed three times with PBS and then cultured in serum-free media for 48 h. The media were collected and cell debris was discarded by gradient centrifugation at 300 g for 10 min and 10,000 g for 30 min at 4°C. Further centrifugation (100,000 g, 4°C, 70 min) was conducted to obtain extracellular vesicles. After resuspending the pellet and washing it with PBS, the pellet was centrifuged at 100,000 g, 4°C, for 70 min. For extracellular vesicle labeling, extracellular vesicles were incubated with 3,3’-dioctadecyloxacarbocyanine perchlorate, DIOC_18_(3) (Beyotime) or 1,1-dioctadecyl-3,3,3,3-tetramethylindotricarbocyanine iodide DIR (Beyotime) at 37°C for 30 min. Samples were then centrifuged at 100,000 g, 4°C, for 70 min (30, 31). EVs were resuspended in PBS and used immediately for experiments. EVs were not frozen and all experiments used freshly prepared EVs. EVs were identified by transmission electron microscopy (H7500; Hitachi, Tokyo, Japan). The same passage numbers of EVs derived from WT and IL-10 knockout MSCs were used for individual experiments.

### Macrophage *In Vivo* Elimination

Clodronate Liposome and Control Liposome were purchased from FormuMax (Palo Alto, CA, USA). C57BL/6 mice were injected intraperitoneally with the indicated liposomes (200 μl/20 g body weight) for 48 h and then underwent the THS procedure (28).

### Flow Cytometry Analysis

NPCs isolated from liver tissue and bone marrow cells were suspended in PBS with 1% BSA (w/v) and then incubated with fluorescent-labeled antibody at 4°C for 30 min. For anti-CD206 staining, the cell membrane was ruptured using Fixation and Permeabilization Solution (BD Cytofix/Cytoperm Plus Kit, 555028). The antibodies used in this study are listed in Supplement table 1. Cells were then gently dispersed in 1% polyoxymethylene (5×10^6^ cells/ml) and subjected to flow cytometry using an CyAn ADP analyzer (Beckman Coulter, Indianapolis, IN, USA). Data were analyzed with the FlowJo software (version 7.6).

### Confocal Microscopy

RAW264.7 cells (1×10^4^) were treated with DIOC18(3)-labeled EVs (20 μg) for 12 h, then incubated overnight with lysosome-specific antibodies (1:1000, Cell Navigator Lysosome Staining Kit, Red Fluorescence, #22658), fixed with 4% paraformaldehyde, and stained with 1 g/ml DAPI for 20 min. Between each step, cells were washed three times with PBS containing 0.5% BSA. Images were acquired with a Leica confocal microscope (Leica, TCSSP8 STED3X). Imaging conditions were maintained at identical settings for each antibody labeling experiment with original gating performed using the negative control.

### *In Vivo* EV Tracking

DiR-labeled EVs (20 μg) were injected into mice *via* the femoral artery. The presence of labeled EVs in liver was detected at 2 or 20 h after injection using a live animal imaging system with excitation at 745 nm and emission at 779 nm.

### Overexpression and Knock Down of PTPN22 in RAW264.7 Cells

The RAW264.7 cell line was obtained from Professor Xiao-Dong Zhang, College of Life Sciences, Wuhan University. The PTPN22 coding sequence was PCR amplified and ligated into the pHAGE vector. The shRNA sequences against PTPN22 were as follows, 5’-CGGCTAAATCAAGCCCTTCTT-3’. We co-transfected 293T cells (from ATCC) cultured in 6-cm petri dishes with the lentiviral packaging vectors psPAX2 (1 μg, Addgene, #12260), pMD2.G (1 μg, Addgene, #12259), and pHAGE-IL-10 (2 μg) to produce lentiviral particles. Three days after transfection, the media were harvested to infect RAW264.7 for 12 h before being replaced with fresh media. Cells were subsequently placed under puromycin selection by addition of puromycin (1 μg/ml). PTPN22 over-expression or knock down were confirmed by western blot.

### Western Blot Analysis

Total protein lysates were extracted from cells or EVs using lysis buffer (Cell Signaling Technology, Beverly, MA, USA) containing complete protease inhibitors (Roche Applied Sciences). Western blotting was performed as previously described (32). The amount of protein loaded in this study is 20 μg. The Western blot membrane were imaged using a ChemiScope 3000 Mini system. The primary and secondary antibodies are listed in Supplement table 1.1.1.

### Quantitative Real-Time PCR

Total mRNA was extracted from liver or cells and reverse transcribed to cDNA using the Revert Aid First Strand cDNA Synthesis Kit (Thermo Fisher Scientific, Waltham, MA, USA). Real-time PCR was performed on the Light Cycler 480 system (Roche, CA, USA) with SYBR Green Master Mix (Takara Biotechnology, Otsu, Shiga, Japan). All reactions were biological replicated and β-actin or GAPDH mRNA served as an internal control. The relative gene expression was calculated using the comparative threshold cycle (Ct) method (relative gene expression =2^-ΔΔCT^). The gene-specific primers are shown in **Supplement Table 1.1.2**.

### RNA-Sequencing Analysis

Isolation of RNA from RAW264.7 cells and RNA-sequencing were performed by Expression Analysis as described previously (33). Trim galore (version 0.4.4) was used for RNA-sequencing analysis. Sequence count normalization, false discovery rate (FDR) and differential gene expression analysis were conducted. Differentially expressed genes were defined by fold change >2 and P <0.05. The PLKO.1 was defined as the control of RAW264.7 cells with knocking down the expression of PTPN22 gene. The shPTPN22 was defined as the RAW264.7 cells with knocking down the expression of PTPN22 gene. The MYC was defined as the control of RAW264.7 cells with PTPN22 gene overexpression.

### Statistical Analysis

All the data in this study are expressed as means ± SD. Comparison of two treatment groups was performed by Student’s t-test. Multiple groups were analyzed by one-way ANOVA analysis. Survival curves were analyzed using the log-rank (Mantel–Cox) test. GraphPad Prism version 8.0 was used for statistical analyses. Statistical significance was considered as p<0.05. In this study, all the replicates were biological replicates (the same experiment repeated in multiple mice).

## Results

### IL-10-Deficient MSCs Lost Protective Capacity in THS-Induced Hepatic Injury

**Figure 1:**
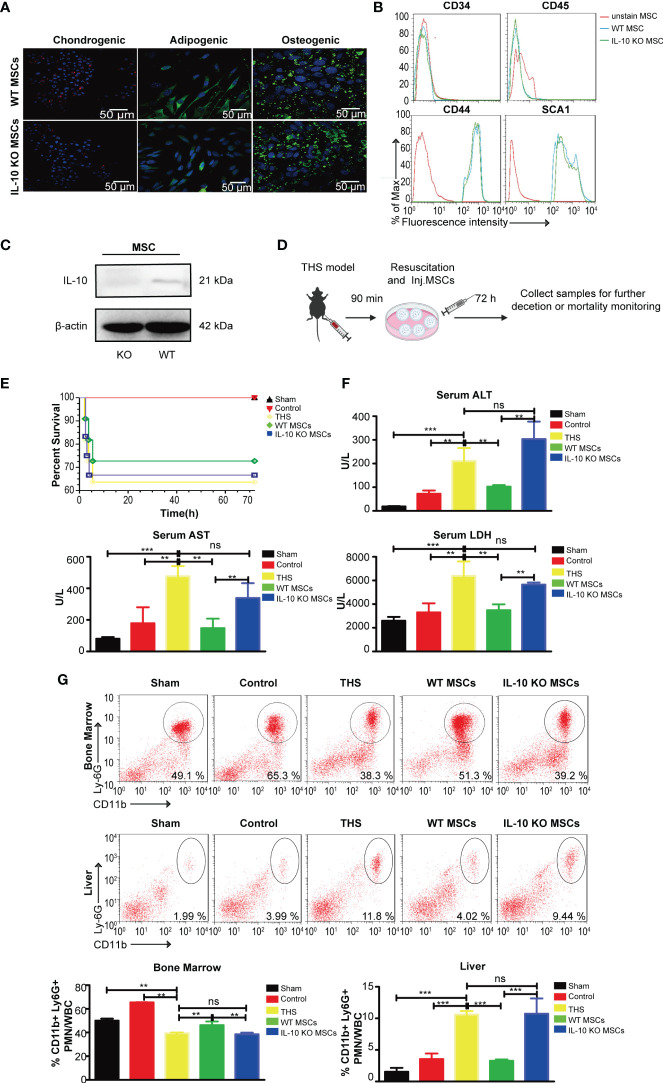
WT MSCs alleviate THS-induced hepatic injury in mice, but IL-10 KO MSCs do not show a protective effect. MSCs and IL-10 KO MSCs were isolated from WT C57/B6 mice and IL-10 KO C57/B6 mice, respectively. **(A)** Representative confocal images showing the adipogenic (FABP4), chondrogenic (Collagen II) and osteogenic (Ostepontin) ability of MSCs and IL-10 KO MSCs. Scale bars, 50 μm. **(B)** Flow cytometry analysis of the surface markers (CD44, SCA1, CD34, CD45) on WT MSCs and IL-10 KO MSCs. **(C)** Western blotting analysis of IL-10 expression in the indicated MSCs (The amount of protein loaded in this study was 20 μg). **(D)** Schematic diagram of the THS model treated by WT MSCs or IL-10 KO MSCs. **(E)** The survival rates of the sham, control, THS, WT MSCs and IL-10 KO MSCs treatment groups; Survival was monitored for 72h after injury (n=10–12). **(F)** Serum LDH, ALT and AST levels in peripheral blood from the indicated groups (n=3–5). **(G)** Flow cytometry analysis of CD11b^+^Ly6G^+^ neutrophils in bone marrow and livers in the indicated groups (n=3–5). **P < 0.01, ***P < 0.001; ns, not significant. All data are shown as the means ± SEM.

To investigate the role of IL-10 in MSC-mediated protection during THS, we first isolated MSCs from the bone marrow of wild-type (WT) and IL-10*^ob/ob^* C57/B6 mice to obtain WT and IL-10 knockout (KO) MSCs, respectively. After 4–5 generations of culture, WT and IL-10 KO MSCs exhibited a fibroblast-like, spindle-shaped appearance and showed adherent growth (**Supplementary Figure 1A**). The pluripotency of the two types of MSCs were verified, and both WT and IL-10 KO MSCs displayed similar differentiation capacities to undergo adipogenesis, chondrogenesis, and osteogenesis, with positive staining of lineage-specific markers FABP4, collagen II and Ostepontin, respectively, after 12 days of induction (**Figure 1A**). Flow cytometry analysis further confirmed that WT and IL-10 KO MSCs showed positive labeling of surface marker proteins (for bone marrow mesenchymal stem cells) Sca-1 and CD44 and negative staining for CD45 and CD34 (**Figure 1B**). IL-10 deletion in IL-10 KO MSCs was confirmed by western blotting (**Figure 1C**).

To explore the effects of MSCs and possible role of IL-10 in THS, we next established a 90 min THS mouse model as described in Methods (**Figure 1D**). Compared with the sham and control mice (only right femur fracture, a localized trauma injury model), the THS model mice displayed a decreased survival rate and hepatic injury, as indicated by the increased serum levels of ALT, AST and LDH (**Figures 1E, F**). Although MSC seems has the trend to improve the mortality of THS mice than IL-10 KO MSC, there was no significant difference between the indicated group (**Figure 1E**). In addition, the serum levels of ALT, AST and LDH in WT MSC–treated THS model mice were lower than levels in THS model mice (**Figure 1F**). The number of neutrophils that emigrated out of bone marrow and infiltrated into the liver tissues was significantly reduced after MSCs treatment (**Figure 1G**). In contrast, treatment with IL-10 KO MSCs had little effect on the survival rate of THS model mice (**Figure 1E**). Furthermore, there was no significant difference in the serum levels of ALT, AST and LDH and the number of infiltrating neutrophils between THS model mice with and without IL-10 KO MSCs treatment (**Figures 1F, G**). These data demonstrate that IL-10 plays an essential role in the protective effect of WT MSCs in THS-induced hepatic injury.

### MSC-EVs Containing IL-10, But Not Exogenous IL-10, Are Important in MSC-Mediated Protection in THS

**Figure 2:**
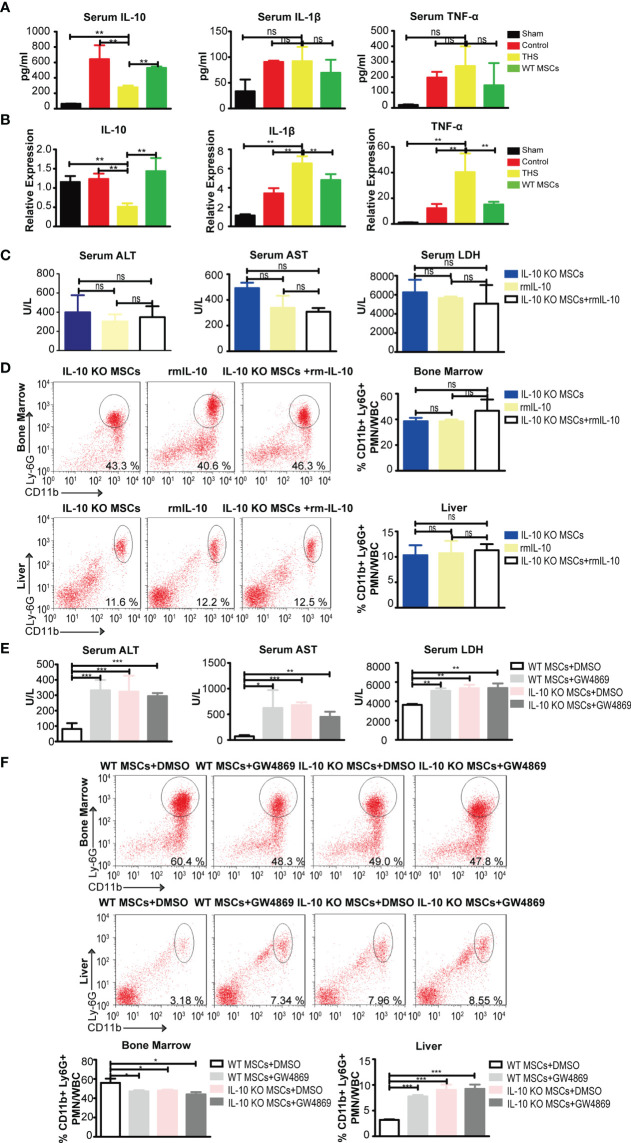
MSC-EVs containing IL-10, but not exogenous recombinant IL-10, exhibit protection against THS in mice. **(A)** ELISA of TNF-α, IL-1β, IL-6 and IL-10 levels in peripheral serum in the indicated groups (n=3–5). **(B)** Quantitative PCR analysis of IL-1 β, IL-6, IL-10 and TNF-α mRNA levels in liver tissues in the indicated groups (n=3–5). **(C, D)** After 90 min of THS, the mice received 5×10^5^ IL-10 KO MSCs or 5×10^5^ IL-10 KO MSCs plus 50 μg/kg recombinant IL-10 *via* the femoral artery at the beginning of resuscitation. **(C)** The serum LDH, ALT and AST levels in peripheral blood were examined in the indicated groups (n=3–5). **(D)** CD11b^+^Ly6G^+^ neutrophils in bone marrow and livers were analyzed in the indicated groups (n=3–5). **(E, F)** 5×10^5^ WT MSCs or 5×10^5^ IL-10 KO MSCs pretreated with GW4869 (5 μg/mL) were infused into mice after THS (n=3–5). The serum levels of LDH, ALT and AST **(E)** and neutrophils in bone marrow and liver tissues **(F)** were measured in the indicated groups (n=3–5). *P < 0.05, **P < 0.01, ***P < 0.001; ns, not significant. All data are shown as the one-way analysis of variance of the mean.

MSCs exhibit a protective role through paracrine actions (34). Previous studies showed that IL-10 secreted by MSCs modulated the systemic inflammatory response induced by brain and myocardial injury (9, 35, 36). To determine if MSCs protect THS-induced hepatic injury through secreting IL-10, we first assessed IL-10 mRNA and protein expression in both peripheral serum and liver tissue of THS model mice. We found that the circulating levels of IL-10 and hepatic IL-10 mRNA significantly increased following injection of MSCs compared with THS model mice (**Figures 2A, B**). In addition, no changes in IL-1β and TNF-α in peripheral serum were observed between THS model mice with or without MSC treatment. We did detect decreased mRNA levels of TNF-α and IL-1β in liver after MSCs treatment compared with THS model mice (**Figures 2A, B**). Based on these results, we hypothesized that MSCs were major sources for the increase of IL-10 in the circulation and local liver tissue.

To determine whether exogenous IL-10 could rescue the abolished protective function in IL-10 KO MSCs, we performed experiments using recombinant mouse IL-10 (rmIL-10). Interestingly, there was no significant difference in serum ALT, AST and LDH levels in THS mice treated with rmIL-10, IL-10 KO MSCs with or control PBS (**Figure 2C**). In addition, no differences were observed in the numbers of neutrophils that emigrated out of bone marrow and infiltrated into the liver (**Figure 2D**). These data suggest that exogenous IL-10 could not rescue the protective effect of MSCs depleted for IL-10, it looks like the IL-10 need some kinds of cargo to implement the function.

To further examine the potential role of EVs in MSC-derived IL-10 mediated protective functions, we pretreated WT or IL-10 KO MSCs with GW4869, an inhibitor of EV biogenesis and release, and then treated THS model mice with these cells. We observed significantly higher levels of serum ALT, AST and LDH in THS mice injected with GW4869-pretreated WT MSCs compared with THS mice injected with WT MSCs (**Figure 2E**). GW4869-pretreatment also blocked the mobilization of neutrophils (**Figure 2F**). GW4869 treatment had no effect on the function of IL-10 KO MSCs in THS-induced hepatic injury. These data strongly implicate the involvement of IL-10-containing EVs in the MSC-mediated protection after THS strike.

### MSC-EVs Alleviate THS-Induced Hepatic Injury in Mice

**Figure 3:**
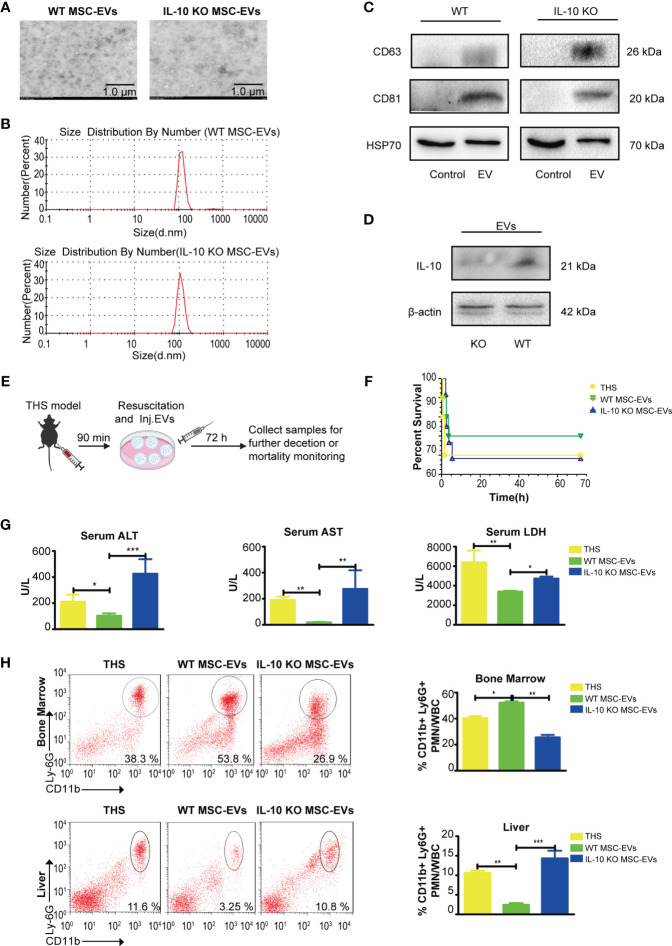
WT MSC-EVs have protective capability in the THS model. **(A)** Identification of WT MSC-EVs and IL-10 KO MSC-EVs by TEM. Scale bar, 1 μm. **(B)** The size distribution of WT MSC-EVs and IL-10 KO MSC-EVs. **(C, D)** Western blot analysis of EV-associated protein markers CD63, CD81 and HSP70 **(C)** and IL-10 expression **(D)** in WT MSC-EVs and IL-10 KO MSC-EVs (The protein loaded of MSC-EVs was 20 μg). **(E)** Schematic diagram of the THS model treated with WT MSC-EVs or IL-10 KO MSC-EVs. **(F–H)** The survival rate of the indicated groups after 72 h resuscitation (n=10–12). **(G)** The plasma levels of LDH, ALT and AST were analyzed in the indicated groups by ELISA (n=3–5). **(D)** Flow cytometry analysis of neutrophils in bone marrow and liver tissues in the indicated groups (n=3–5). *P<0.05, **P<0.01, ***P<0.001. All data are shown as the one-way analysis of variance of the mean.

To further examine whether MSCs-derived IL-10 are encapsulated in EVs and transmitted to targeted cells, we next isolated WT and IL-10 KO EVs by ultracentrifugation. There was no difference in morphology between the two types of EVs under transmission electron microscope (TEM) (**Figure 3A**). The diameters of the two types of EVs ranged from 90 to 142 nm, with a mean diameter of 116 nm (**Figure 3B**). Western blotting assay further confirmed the expression of EV-associated proteins CD63 and CD81 in the two types of EVs (**Figure 3C**) and the disappearance of IL-10 in IL-10 KO MSC-EVs (**Figure 3D**).

We next evaluated the therapeutic efficacy of the two types of EVs. WT MSC-EVs or IL-10 KO MSC-EVs were infused into THS mice at the resuscitation phase (**Figure 3E**). Although THS treated with WT MSC-EVs seems to have the trend to improve the mortality than the group treated with IL-10 KO MSC-EVs, there was no significant difference between the indicated group. WT MSC-EV treatment significantly alleviated THS-induced hepatic injury, as indicated by the decreased serum levels of ALT, AST and LDH in peripheral serum, compared with THS mice, whereas IL-10 KO MSC-EVs did not show these protective effects (**Figures 3F, G**). In addition, the number of neutrophils that emigrated out of bone marrow and infiltrated into liver tissues was significantly reduced after WT MSC-EVs treatment compared with IL-10 KO MSC-EVs treatment (**Figure 3H**). Together, these data demonstrated a crucial role of IL-10-containing EVs in MSC-mediated protection against THS-induced hepatic injury.

### The Infused MSC-EVs Were Taken up by Kupffer Cells

**Figure 4:**
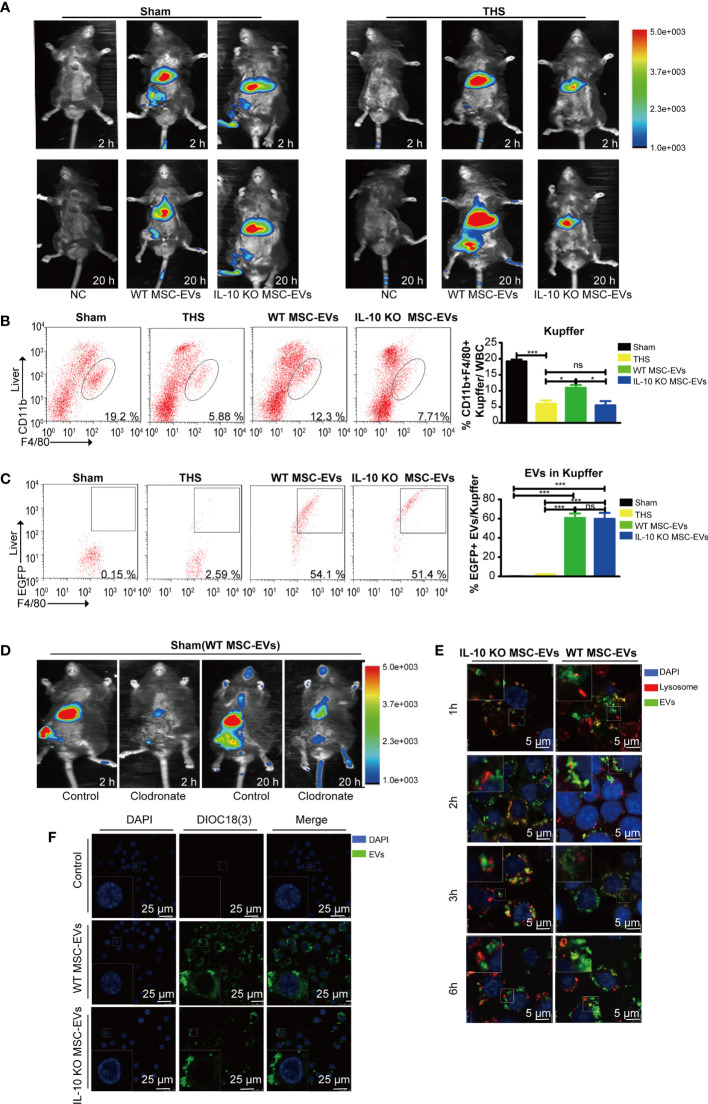
MSC-EVs protect THS-induced hepatic injury by interacting with Kupffer cells. **(A)** The *in vivo* distribution of injected EVs labeled with DiR into the sham or THS mice at 2 h and 20 h, respectively. **(B, C)** Flow cytometry analysis of the number of CD45^+^F4/80^+^CD11b^int^ Kupffer cells (framed, B) and DIOC_18_(3)-positive cells in the above CD45^+^F4/80^+^CD11b^int^ Kupffer cells **(C)** in the indicated groups (n=3–5). **(D)** Clodronate was used to specifically delete macrophages. DiR-labeled EVs were infused into mice and tracked by *in vivo* imaging at 2 h and 20 h, respectively. **(E)** Representative confocal images of the subcellular localization of EVs in RAW264.7 cells. Scale bars, 25 μm. **(F)** Co-localization of EVs with lysosomes in RAW264.7 cells. Scale bars, 5 μm. *P < 0.05, ***P < 0.001; ns, not significant. All data are shown as the means ± SEM.

To investigate the organ target by MSC-EVs, we tracked DiR-labeled EVs *in vivo* in THS-stressed mice. We found that the majority of infused WT MSC-EVs and IL-10 KO MSC-EVs were distributed in the liver area at 2 h and 20 h after resuscitation (**Figure 4A**), indicating that the liver tissues were direct targets of MSC-EVs after THS. Notably, we observed strongly reduced aggregation of IL-10 KO MSC-EVs in liver tissues after THS compared with WT MSC-EVs. However, the accumulation of IL-10 KO MSC-EVs in liver tissues showed no difference with sham-operated mice (**Figure 4A**). All these results indicated EVs were mostly uptake by the liver.

Liver macrophages are the major cells that engulf exogenous particles, including EVs. Thus, we analyzed the number of F4/80^+^CD11b^int^ macrophages (Kupffer cells) in liver tissues by flow cytometry. The number of positively stained cells was suppressed by THS in the model mice, but was augmented in THS mice injected with WT MSC-EVs. However, the effect was diminished in THS mice injected with IL-10 KO MSC-EVs (**Figure 4B**). These results indicate IL-10 of MSC-EVs directly affected the number or functions of Kupffer cells after THS. To further confirm the role of Kupffer cells in EV function, we labeled EVs with DIOC_18_(3) and injected these into sham- or THS-operated mice. Flow cytometry revealed that Kupffer cells showed staining for DIOC_18_(3)-labeled EVs. In addition, there were no significant differences in the number of double-positive cells in livers from THS mice injected with WT or IL-10 KO MSC-EVs (**Figure 4C**), suggesting a comparable capacity of liver macrophages to uptake WT or IL-10 KO MSC-EVs.

To further illustrate the important role of macrophages in EV-mediated protection, we deleted macrophages *in vivo* by intraperitoneal injection of clodronate liposomes into mice 2 days before the THS procedure. Flow cytometry confirmed a loss of macrophages in the liver and lung tissues after clodronate injection (**Supplementary Figure 2A**). Depletion of systemic macrophages led to a significant EV reduction in the liver of sham-operated mice, particularly at 2 h (**Figure 4D**). To evaluate the IL-10+ MSC-EVs effect of macrophage deprivation on THS-induced hepatic injury, we examined survival rate and ALT, AST and LDH serum levels. No significant differences were observed in survival or serum markers among THS mice, WT MSC-EV–injected THS mice or IL-10 KO MSC-EV–injected THS mice after the deletion of systemic macrophages (**Supplementary Figure 2B**). Moreover, confocal microscopy was performed to determine the sub-cellular localization of MSC-EVs in RAW264.7 cells. The results revealed that MSC-EVs and IL-10 KO MSC-EVs were taken up by RAW264.7 cells and showed cytoplasmic localization (**Figure 4F**). We next examined whether EVs were transported to lysosomes for degradation using confocal microscopy. Only a small portion of EVs colocalized with lysosomes in RAW264.7 cells, indicating that while some EVs were degraded by lysosomes, most EVs delivered IL-10 cargos to function in Kupffer cells (**Figure 4E**). IL-10 KO MSC-EVs tended to accumulate to higher levels in lysosomes, suggesting increased degradation of these EVs (**Figure 4E**). Together, these findings indicate that MSC-EVs exert their protective function in THS *via* macrophages, and IL-10 deletion modulated EV degradation by lysosomes without affecting their uptake by macrophages.

### WT MSC-EVs Promote Anti-Inflammatory Polarization of Kupffer Cells

**Figure 5:**
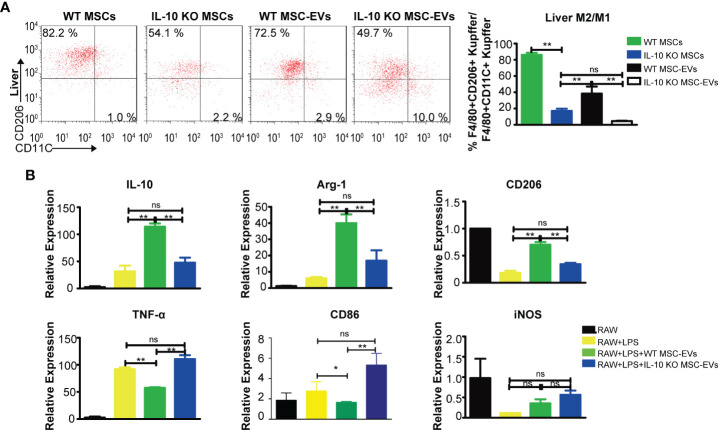
WT MSC-EVs promote the anti-inflammatory polarization of macrophages. **(A)** Flow cytometry analysis of the proportion of CD45^+^CD11b^+^F4/80^+^CD11C^+^ and CD45^+^CD11b^+^F4/80^+^CD206^+^ cells in liver tissues from THS mice treated with the indicated MSCs (5×10^5^) or EVs (20 μg) (n=3–5). **(B)** RAW264.7 cells pretreated with 100 ng/ml LPS for 12 h were exposed to the indicated EVs (20 μg) for another 12 h The mRNA levels of pro-inflammatory markers (TNF-α, CD86, CD11C) and anti-inflammatory markers (IL-10, CD206, Arg-1) were detected by RT-PCR. *P < 0.05, **P < 0.01; ns, not significant. There were three samples (individual biological sample) in each group. All data are shown as the means ± SEM.

Macrophages undergo specific differentiation into distinct functional phenotypes depending on the local tissue environment (37, 38). To determine whether IL-10 as cargo in EVs influences the balance between pro-inflammatory phenotypes and anti-inflammatory phenotypes of Kupffer cells after THS strike, we used flow cytometry to evaluate pro-inflammatory and anti-inflammatory macrophages in liver from THS mice treated with MSCs and MSC-EVs. WT MSCs and WT MSC-EVs dramatically increased the number of anti-inflammatory Kupffer cells and decreased the number of pro-inflammatory Kupffer cells compared with IL-10 KO MSCs and IL-10 KO MSCs groups (**Figure 5A**). These results indicate IL-10 from MSC-EVs as an important inducer of anti-inflammatory polarization of Kupffer cells.

Next, RAW264.7 cells were stimulated with LPS to mimic the *in vivo* inflammation response. To evaluate macrophage polarization, we examined the gene expressions of anti-inflammatory markers (IL-10, Arg-1, CD206) and pro-inflammatory markers (TNF-α, CD86, iNOS). MSC-EVs induced a remarkable increase in anti-inflammatory genes (IL-10, Arg-1, CD206) and a decrease in pro-inflammatory genes (TNF-α, CD86), whereas IL-10 deficiency in MSC-EVs led to lower levels of anti-inflammatory markers and higher levels of pro-inflammatory markers. The levels of iNOS were not significantly altered by MSC-EVs (**Figure 5B**). These findings indicated that MSC-EVs interact with and promote the anti-inflammatory polarization of liver macrophages through the delivery of IL-10 as cargo.

### WT MSC-EVs Promote the Anti-Inflammatory Polarization of Macrophage *via* PTPN22

**Figure 6:**
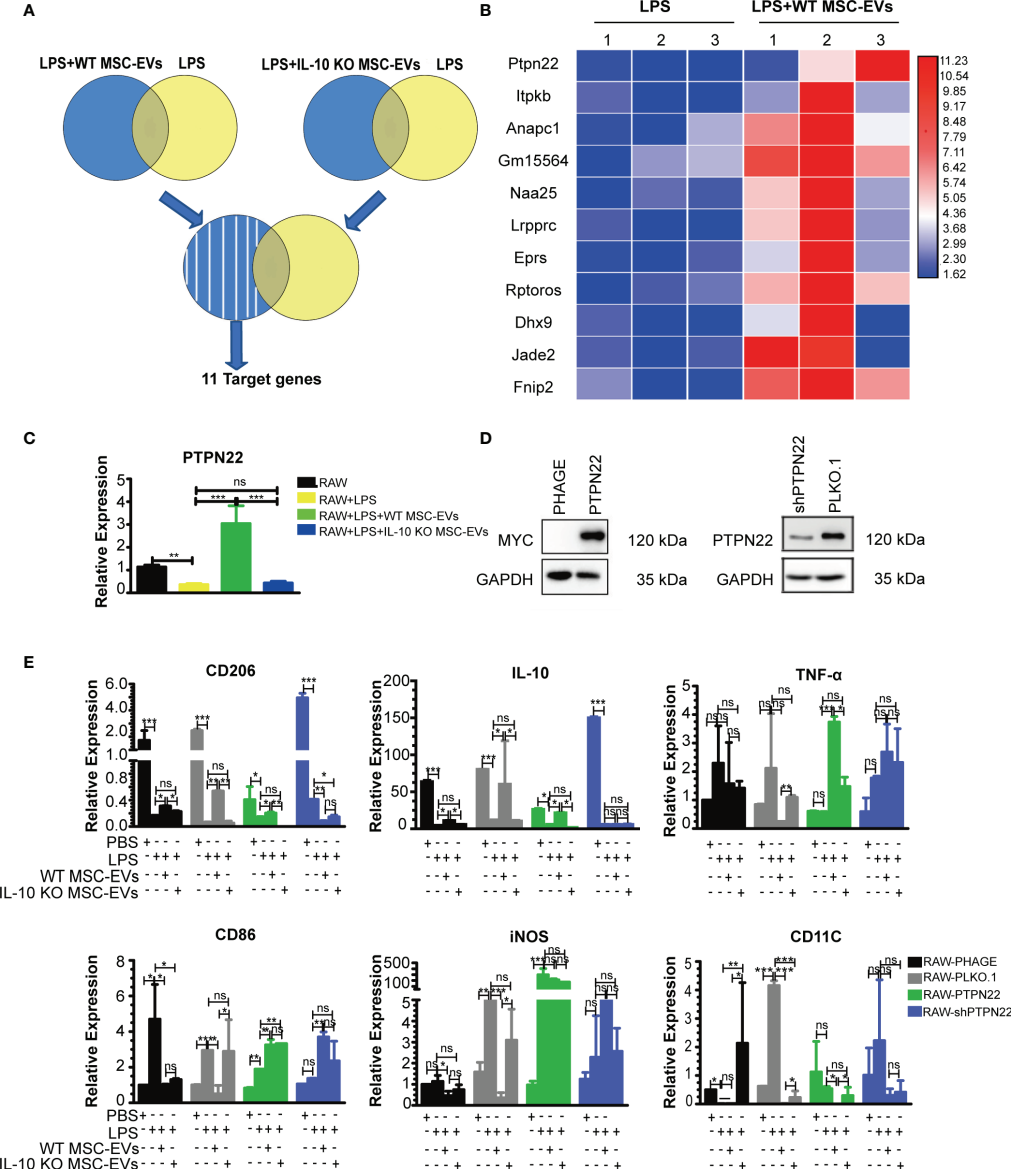
PTPN22 is important for WT MSC-EV-mediated anti-inflammatory polarization of RAW264.7 cells. **(A)** Experiment outline: RAW264.7 cells were pre-exposed to LPS (100 ng/ml) for 12 h, treated with EVs for either WT or IL-10 KO MSCs for another 12 h, and subjected to RNA sequencing. Differentially expressed genes (Log |FC|≥2 and P < 0.05) were analyzed. **(B)** Heat map screen of the top 11 differential genes according to transcriptome sequencing. **(C)** RT-PCR analysis of the mRNA level of PTPN22 in RAW264.7 cells treated with the indicated groups. **(D)** Western blotting analysis of PTPN22 in RAW264.7 cell lines with overexpression of PTPN22 (phage-PTPN22) and its control (phage-MYC) (left panel); as well as in RAW264.7 cell lines with knocking down the expression of PTPN22 (shPTPN22) and its control (PLKO.1) (The amount of protein loaded is 20 μg). The PLKO.1 was defined as the control of RAW264.7 cells with knocking down the expression of PTPN22 gene. The shPTPN22 was defined as the RAW264.7 cells with knocking down the expression of PTPN22 gene. The MYC was defined as the control of RAW264.7 cells with PTPN22 gene overexpression. **(E)** PTPN22 over-expressing or down-regulated RAW264.7 cells were exposed to LPS for 12 h and then treated with 20 μg of MSC-EVs or IL-10 KO MSC-EVs for another 12 h The mRNA levels of pro-inflammatory markers (TNF-in CD86, iNOS, CD11C) and anti-inflammatory markers (IL-10, CD206) were detected by RT-PCR. *P < 0.05, **P < 0.01, ***P < 0.001; ns, not significant. There were three samples (individual biological sample) in each group. All data are shown as the means ± SEM.

To further clarify the functional mechanism of IL-10+ MSC-EVs in liver macrophage polarization after THS strike, we examined the genes regulated by MSC-EVs by transcriptome sequencing. RAW264.7 cells were pre-exposed to 100 ng/ml LPS for 12 h, treated with EVs for another 12 h, and subjected to RNA sequencing. Differentially expressed genes (Log |FC|≥2 and P < 0.05) were then analyzed. The differentially expressed genes in cells treated with MSC-EVs compared with controls and the differentially expressed genes in cells treated with IL-10 KO MSC-EVs compared with controls were identified (**Figure 6A**). The common genes between the differentially expressed genes in cells treated with MSC-EVs and those in cells treated with IL-10 KO MSC-EVs were removed, as these are likely regulated by components of EVs other than IL-10. The remaining 11 genes were considered to be regulated by IL-10 in MSC-EVs.

Protein tyrosine phosphatase non receptor 22 (PTPN22) showed the most significant increase in expression in the MSC-EVs group compared with the RAW+LPS group (**Figure 6B**). A previous study showed that PTPN22 negatively regulates pro-inflammatory macrophage activation (39), which was consistent with the outcome of MSC-EV-treated macrophages. Quantitative PCR analysis showed that LPS suppressed the expression of PTPN22 mRNA, which was induced by MSC-EVs but did not show changes following IL-10 KO MSC-EV treatment compared with controls (**Figure 6C**).

To clarify the function of PTPN22 on macrophage polarization, we constructed RAW264.7 cell lines that stably overexpressed or downregulated PTPN22 (**Figure 6D**). Then we examined anti-inflammatory and pro-inflammatory markers after EVs treatment. We observed a higher level of anti-inflammatory markers (IL-10, CD206) in the presence of overexpressed PTPN22 treated with IL-10+ EVs and lower levels of anti-inflammatory markers (IL-10, CD206) in the presence of overexpressed PTPN22 treated with IL-10- EVs in the RAW264.7 cells upon the stimulation of LPS. Whereas, the trend of pro-inflammatory markers CD11C were the opposite of these anti-inflammatory markers in the RAW264.7 cells upon the stimulation of LPS. Somehow, there was not significant difference between IL-10- EVs and IL-10+ EVs treated group for the expression of anti-inflammatory (IL-10, CD206) and pro-inflammatory (CD11C) markers in the PTPN22 downregulated RAW264.7 cells upon the stimulation of LPS (**Figure 6E**). Indicating that PTPN22 plays a key role during inflammatory response in our THS model with the treatment of EVs.

To our surprise, the trends of the other 3 pro-inflammatory markers (TNF-α, CD86, iNOS) were not always consistent with CD11C. In PTPN22 overexpressed RAW264.7 cells, the expression of CD86 and TNF-α were significantly increased in EVs treated group (both IL-10- EVs and IL-10+ EVs) compared with the control upon the stimulation of LPS. Conversely, IL-10+ EVs led a higher level of TNF-α in PTPN22 overexpressed RAW264.7 compared with PTPN22 overexpressed RAW264.7 treated with IL-10- EVs upon the stimulation of LPS. Interestingly, the expression of iNOS had no difference between EVs treated group (both IL-10- EVs and IL-10+ EVs) with control group in PTPN22 overexpressed RAW264.7 cells upon the stimulation of LPS. Moreover, there was no significant difference in the other 2 pro-inflammatory markers (TNF-α and iNOS) in EVs treated group (both IL-10- EVs and IL-10+ EVs) compared with the control group in PTPN22 downregulated RAW264.7 cells upon the stimulation of LPS. In comparison with control, IL-10+ EVs, not IL-10- EVs treatment led a significant higher level of CD86 in PTPN22 down regulated RAW264.7 cells upon the stimulation of LPS (**Figure 6E**). Taken together, these results suggested that the PTPN22 enriched the anti-inflammatory signals such as IL-10 and CD206 as well as pro-inflammatory signals, such as CD11C during the polarization process of RAW264.7 cells after suffering the THS injury, whereas has limited effect on other pro-inflammatory associated signaling pathway e.g., TNF-α, CD86, iNOS.

## Discussion

Several mouse hemorrhagic shock (HS) models have been established, including fixed-volume, fixed-pressure, and uncontrolled hemorrhage models, with or without tissue injury. In this study, we used the fixed-volume model, which has features of repeatability and easy control; in addition, this model adequately reflects the clinical pathophysiology and treatment of shock in patients (40). In our model, a fixed volume of blood (up to 50% of the total blood volume) was withdrawn from the anesthetized animal. After THS stress, the animals were monitored or resuscitated. When developing a THS model, the time points of hemorrhage, resuscitation and sample collection should be carefully considered. However, these time points have not been consistent among previous reports. Some researchers reported that the most severe organ injury occurred at 90 min in HS. Most previous reports assessed the biochemical markers of organ injury in the 6–24 h after resuscitation (41, 42). However, immune disorder and recovery can occur near 72 h of trauma (43). Therefore, in our HS model, the animals were hemorrhaged for 90 min and the parameters were detected after 72 h of HS. We found that most mice died of THS within 6 h of resuscitation, which was probably due to the deterioration of physiological dysfunction with a cytokine storm phenomenon (44).

MSCs have attracted considerable attention in various fields of medicine (45). However, research on the safety and effectiveness of the clinical application of MSCs is still in the early stages and many obstacles remain to be addressed, such as how to optimize the reparative potential of MSCs, how to regulate their abnormal proliferation and tumorigenesis ability, and how to regulate the relationship between MSCs and other immune cells (46–48). Potter found that MSC and MSC-EVs attenuate lung injury after THS by regulating the GTPase RhoA (49). A recent report identified liver Kupffer cells as the primary target cells of EVs and other exogenous nanovectors (50), which is consistent with our findings that MSC-EVs targeted hepatic Kupffer cells. We further found that the function of MSCs in THS is dependent on IL-10 contained EVs, while free IL-10 had no positive influence on THS. In addition, IL-10-containing EVs from MSCs suppressed the inflammatory response and contributed to recovery of liver function after THS.

EVs are small membrane-bound vesicles that carry cargo, such as RNAs and proteins, and play important roles in the regulation of the immune response, inflammatory cascade, and tumor development (51–53). In this study, we detected an increased anti-inflammatory polarization of macrophages following MSC-EV treatment and found that IL-10 played a critical role in promoting polarization. Our future studies will assemble and modify MSC-EVs containing IL-10 *in vitro* to target treatment and improve THS-induced organ damage.

IL-10 is the most potent anti-inflammatory cytokine induced under stress conditions (54). Before our intervention experiments, we first investigated the effect of different dose (10 and 50 μg/kg) of rmIL-10 on THS-induced liver injury. As indicated by ALT levels, we observed that both 10 and 50 μg/kg rmIL-10 treatment cannot alleviate THS-induced liver injury, and there was no difference between 10 and 50 μg/kg rmIL-10 treated groups. The results indicated that rmIL-10 only induce limited biological responses in our model. IL-10 has a short half-life, with a mean terminal phase half-life of 2.7 to 4.5 h (55). Human clinical trials have examined the use of recombinant IL-10 to treat inflammatory diseases for over a decade. However, exogenous IL-10 has been shown to be a largely ineffective therapy (56). The effect of IL-10 is highly variable depending upon many factors, such as short half-life, the route of administration, treatment time, animal age, or gender, suggesting the potential role of why exogenous IL-10 does not work in this THS model at the timepoint of 72 hours post-surgery.

EVs could be a carrier with complex cargoes including proteins, lipids, and nucleic acids, which confer stability and can direct their cargoes to specific cell types. Liu et al. has reported that EVs is a promising delivery platform to manipulate IL-10 for the effective treatment of ischemic acute kidney injury (24). Our current data indicated that the function of MSCs in THS is dependent on IL-10 contained EVs, while exogenous IL-10 had no positive influence on THS. In addition, IL-10-containing EVs from MSCs suppressed the inflammatory response and contributed to recovery of liver function after THS. Taken together, the mechanism of IL-10 from MSC-EVs or exogenous IL-10 during THS injury in this study is different. However, more in-depth studies are needed to confirm how EVs play a role after being up-taken by macrophage, as well as understand how MSC-EVs carrying IL-10 as a cargo, which might be the direction of our future study.

**Figure 7:**
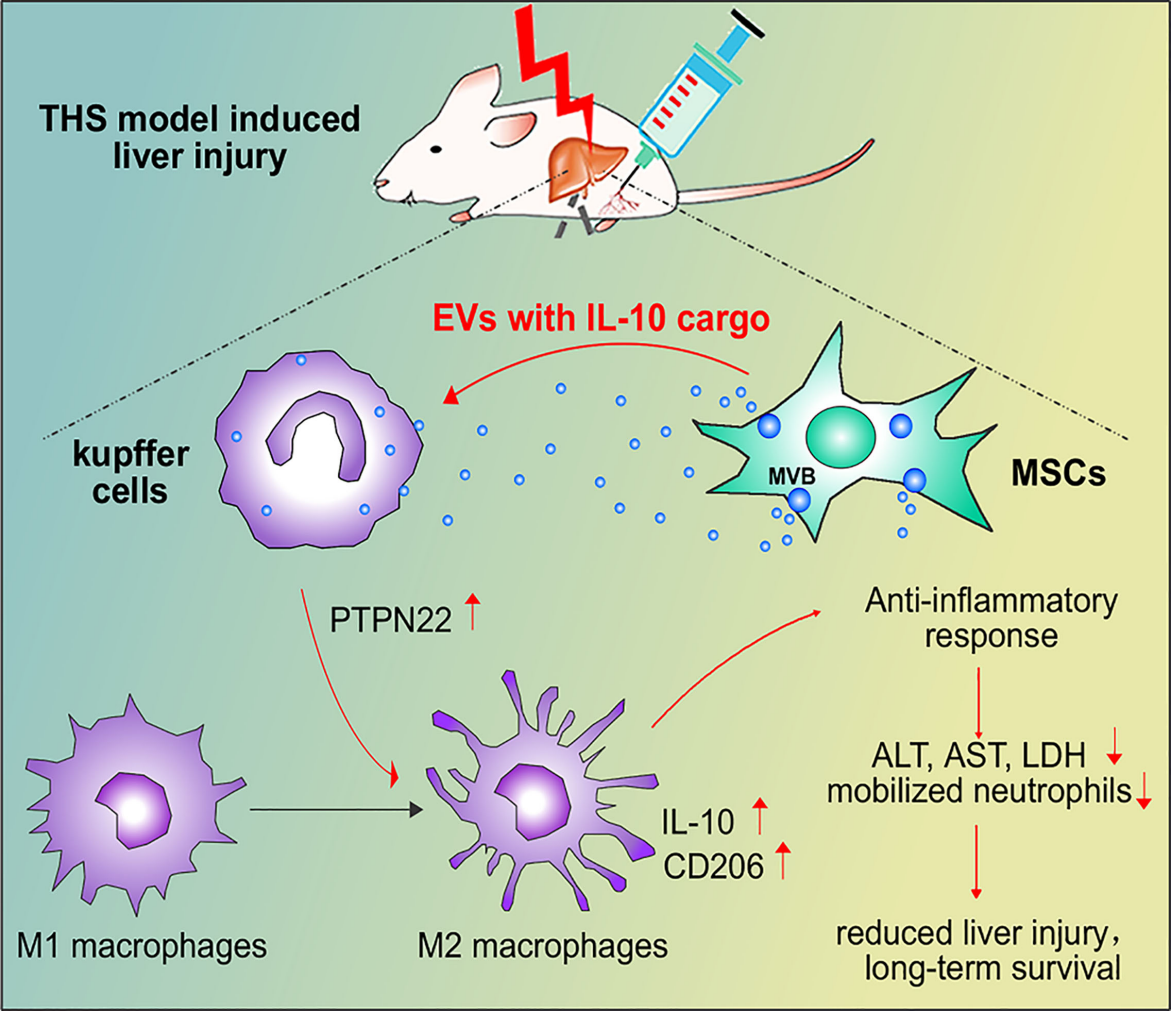
Schematic model of the interactions among MSC-EVs, IL-10, PTPN22 and Kupffer cell polarization after THS. After THS, MSC-EVs primarily accumulate in liver tissues, where they are taken up by Kupffer cells. MSC-EVs carrying IL-10 induce the expression of PTPN22, IL-10 and CD206 and promote the anti-inflammatory polarization of Kupffer cells, which further reduces the plasma levels of LDH, ALT and AST, decreases liver neutrophil infiltration, and restricts neutrophil mobilization from bone marrow during THS. These effects consequently reduce THS-induced hepatic injury and prolong animal survival.

In conclusion, our results showed that IL-10+ MSC-EVs are taken up by Kupffer cells to increase the expression of PTPN22 and promote anti-inflammatory polarization of macrophages after THS. However, in when PTPN22 was decreased in RAW cells, MSCs-EVs lost these protective effects. IL-10 contained in MSC-EVs, but not free IL-10, was indispensable for anti-inflammatory polarization of Kupffer cells (**Figure 7**). Overall, our study implicates MSCs and MSC-EVs in THS and provides new insights into the clinical application of IL-10-containing EV–mediated immunosuppression.

## Data Availability Statement

The datasets presented in the study are publicly available. This data can be found here: https://www.ncbi.nlm.nih.gov/bioproject/PRJNA786410.

## Ethics Statement 

The animal study was reviewed and approved by Animal Care and Use Committee of Wuhan Union Hospital.

## Author Contributions

JZ and HW conceived and designed the experiments. YZ, PS, PX, and WP performed the experiments. PS and ZW bred the animals. YZ, XFZ, HZ, and PS analyzed the data and drafted the article. HZ, GW, KH, and PH participated in data interpretation. XDZ made valuable suggestions for the experiments. JZ and HW critically revised the article and supervised the whole project. YZ, XFZ, HZ, and PS have contributed equally to this work. All authors contributed to the article and approved the submitted version.

## Funding

This study was supported by grants from the National Natural Science Foundation of China under grant (no.81700558, no.81570570, no.81801923, no.81670575 and no.81070355); Pre-Research Fund for Free Innovation of Union Hospital, Huazhong University of Science and Technology under grant (no.02.03.2017-59, no. 02.03.2017-312 and no.02.03.2018-126) and Ningxia Hui Autonomous Region’s key and projects attract talent under grant (no.2018BEB04028).

## Conflict of Interest

The authors declare that the research was conducted in the absence of any commercial or financial relationships that could be construed as a potential conflict of interest.

## Publisher’s Note

All claims expressed in this article are solely those of the authors and do not necessarily represent those of their affiliated organizations, or those of the publisher, the editors and the reviewers. Any product that may be evaluated in this article, or claim that may be made by its manufacturer, is not guaranteed or endorsed by the publisher.
