# Supplementary material for: Mesenchymal Stem Cells Derived Extracellular Vesicles Alleviate Traumatic Hemorrhagic Shock Induced Hepatic Injury via IL-10/PTPN22-Mediated M2 Kupffer Cell Polarization
Source: Front Immunol. 2022 Jan 12;12:811164. doi: 10.3389/fimmu.2021.811164 (PMC8790700; doi:10.3389/fimmu.2021.811164)
Supplement: Supplementary file 1 [file DataSheet_1.docx]

Supplementary Material

# Supplementary Figures and Tables

## Supplementary Table

### Supplement Table 1. All antibodies used in immunocytochemistry, Flow cytometry and Western blots.

| **ID** | **Antibody** | **Company and Catalog** |
| --- | --- | --- |
| 1 | anti-CD34-FITC | BD Biosciences，clone RAM34 |
| 2 | anti-CD45-Pacific Blue | Biolegend, clone 30-F11 |
| 3 | anti-CD44-APC-Cy7 | BD Biosciences, clone IM7 |
| 4 | anti-SCA1-PE | BD Biosciences, clone D7 |
| 5 | anti-CD11b-PE-Cy7 | Biolegend, clone M1/70 |
| 6 | anti-F4/80-PerCP-Cy5.5 | Biolegend, clone BM8 |
| 7 | anti-Ly6G-PE | BD Biosciences, clone 1A8 |
| 8 | anti-CD11C-APC | BD Biosciences, clone HL3 |
| 9 | anti-CD206-PE | BD Biosciences, clone MR5D3 |
| 10 | polyclonal anti-IL-10 antibody | Abclonal, #A2171 |
| 11 | polyclonal anti-β-actin antibody | CELL SIGNALING TECHNOLOGY, #3700 |
| 12 | polyclonal anti- CD63 antibody | CELL SIGNALING TECHNOLOGY, #55051 |
| 13 | polyclonal anti- CD81 antibody | CELL SIGNALING TECHNOLOGY, #10037 |
| 14 | polyclonal anti- HSP70 antibody | CELL SIGNALING TECHNOLOGY, #4873 |
| 15 | polyclonal anti- PTPN22 antibody | CELL SIGNALING TECHNOLOGY, #14693S |
| 16 | polyclonal anti- GAPDH antibody | CELL SIGNALING TECHNOLOGY, #97116 |
| 17 | lysosome-specific antibodies | Cell Navigator Lysosome Staining Kit, #22658 |

### Supplement Table 2. The gene-specific primers of quantitative real-time PCR.

| **ID** | **Primer name** | **Primer sequence (5'to3')** |
| --- | --- | --- |
| 1 | β-actin forward | CAGATGCCACTACAGCACG |
| 2 | β-actin reverse | CCTGCCGCTGCCATAGAAG |
| 3 | IL-1β forward | GGATGAGGACATGAGCACCT |
| 4 | IL-1β reverse | GGAGCCTGTAGTGCAGTTGT |
| 5 | IL-6 forward | AGTTGCCTTCTTGGGACTGA |
| 6 | IL-6 reverse | TCCACGATTTCCCAGAGAAC |
| 7 | IL-10 forward | CCCTTTGCTATGGTGTCCTT |
| 8 | IL-10 reverse | TGGTTTCTCTTCCCAAGACC |
| 9 | TNF-α forward | ACTGAACTTCGGGGTGATCG |
| 10 | TNF-α reverse | GGCTACAGGCTTGTCACTCG |
| 11 | CD206 forward | GGAAACGGGAGAACCATCAC |
| 12 | CD206 reverse | GGCGAGCATCAAGAGTAAAG |
| 13 | Arg-1 forward | CTCCAAGCCAAAGTCCTTAGAG |
| 14 | Arg-1 reverse | AGGAGCTGTCATTAGGGACATC |
| 15 | CD86 forward | ACGGACTTGAACAACCAGAC |
| 16 | CD86 reverse | TGCAGTCCCATTGAAATAAG |
| 17 | iNOS forward | CCAAGCCCTCACCTACTTCC |
| 18 | iNOS reverse | GGCAGTGTAACTCTTCTGCAT |
| 19 | CD11C forward | CCGATCACTCTTCGCCTCAA |
| 20 | CD11C reverse | GAGGCCCATCATGCTGAGAT |
| 21 | PTPN22 forward | CGGTAGAAGCTGACTCTTGTCC |
| 22 | PTPN22 reverse | CCAACTCTTCCTCGGCATTCATC |
| 23 | GAPDH forward | GGTGAAGGTCGGTGTGAACG |
| 24 | GAPDH reverse | CTCGCTCCTGGAAGATGGTG |

## Supplementary Figure

##
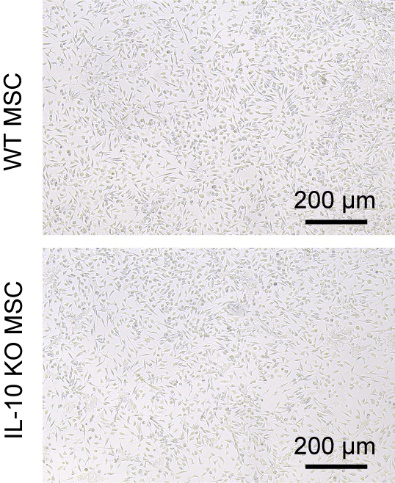


**1.2.1 Supplementary Figure 1.** The morphology of wild-type (WT) MSCs and IL-10 knockout (KO) MSCs under optical microscope. Scale bar, 200 μm.


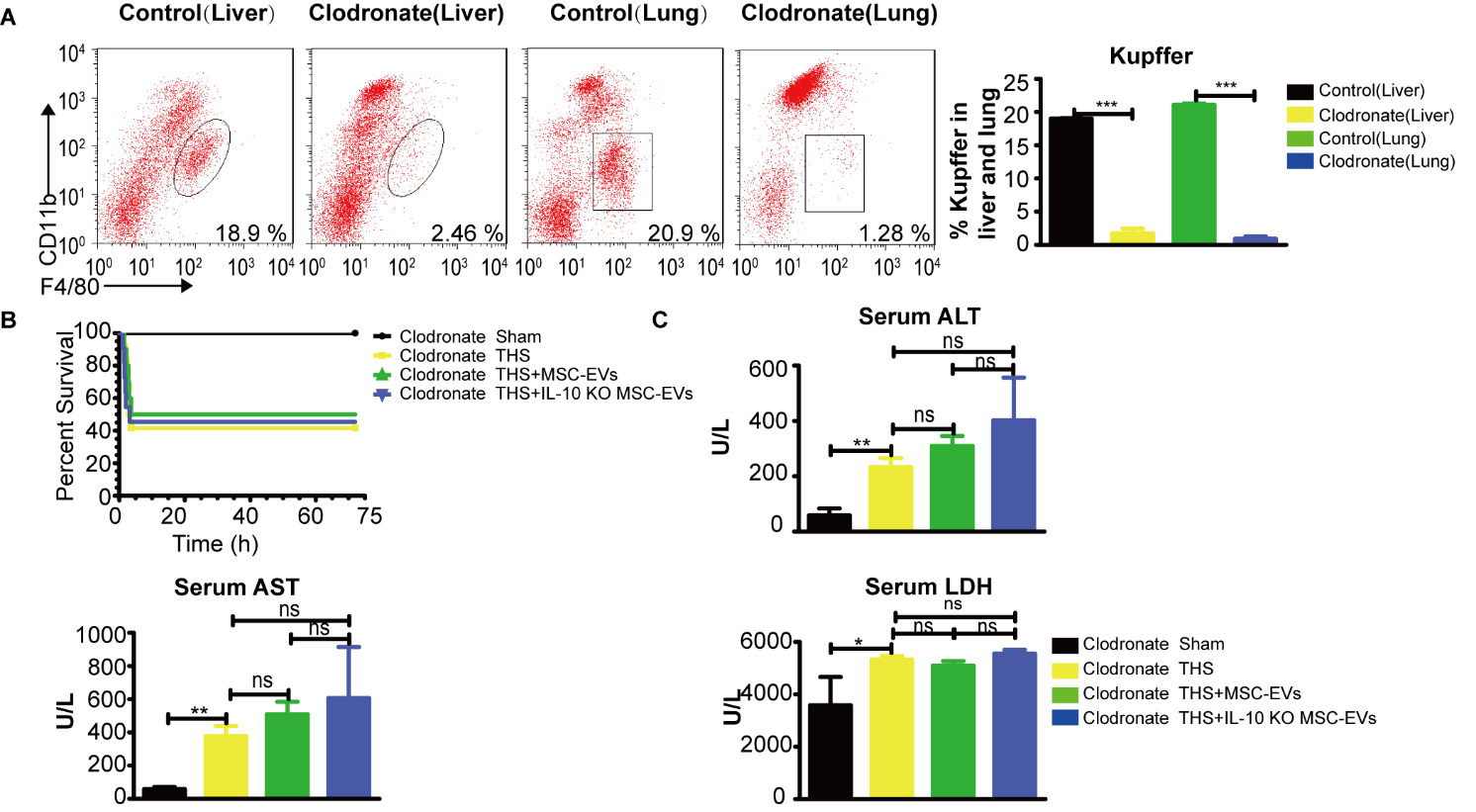


**1.2.2 Supplementary Figure 2.** **Macrophages are essential for the protective effect of WT MSC-EVs on THS-induced liver injury.** (A) The flow cytometry was performed 48 hours after clodronate administration. Flow cytometry analysis of the number of macrophages in liver and lung tissues from mice treated with the indicated liposomes (left panel) (n=3-5). Quantitative analysis of flow cytometry (right panel). (B) The survival rate of the indicated groups after 72 h resuscitation (n=10–12). (C) The plasma levels of LDH, ALT and AST in the indicated groups (n=10–12). *P<0.05, **P<0.01, ***P<0.001. ns, no significant. All data are shown as the mean ± standard error of the mean.
